# Efficacy and safety of navigation robot-assisted versus conventional oblique lateral lumbar interbody fusion with internal fixation in the treatment of lumbar degenerative diseases: A retrospective study

**DOI:** 10.1097/MD.0000000000039261

**Published:** 2024-08-09

**Authors:** Min Tong, Siping Zhang, Wenhao Zhang, Limin Mou, Zhenyu Dong, Rong Wang, Shida Li, Yifei Huang

**Affiliations:** a Department of Spinal Surgery, Traditional Chinese Medicine Hospital Affiliated to Xinjiang Medical University, Urumqi, P.R.China; b Department of Spinal Surgery, Xinjiang Uygur Autonomous Region Hospital of Traditional Chinese Medicine, Urumqi, P.R.China; c Xinjiang Uygur Autonomous Region Academy of Traditional Chinese Medicine, Urumqi, P.R.China.

**Keywords:** fluoroscopy, lumbar degenerative disease, navigation robot, oblique lateral lumbar interbody fusion, percutaneous pedicle screw internal fixation

## Abstract

Effective internal fixation with pedicle screw is a key factor in the success of lumbar fusion with internal fixation. Whether navigation robots can improve the efficacy and safety of screw placement is controversial. Thirty-eight patients who underwent oblique lateral lumbar interbody fusion internal fixation from March 2022 to May 2023 were retrospectively analyzed, 16 cases in the navigational robot group and 22 cases in the fluoroscopy group. Using visual analog score (VAS) for the low back and lower limbs, Oswestry Disability Index to compare the clinical efficacy of the 2 groups; using perioperative indexes such as the duration of surgery, intraoperative blood loss, intraoperative fluoroscopy times, and postoperative hospital stay to compare the safety of the 2 groups; and using accuracy of pedicle screws (APS) and the facet joint violation (FJV) to compare the accuracy of the 2 groups. Postoperative follow-up at least 6 months, there was no statistically significant difference between the 2 groups in the baseline data (*P* > .05). The navigational robot group’s VAS-back was significantly lower than the fluoroscopy group at 3 days postoperatively (*P* < .05). However, the differences between the 2 groups in VAS-back at 3 and 6 months postoperatively, and in VAS-leg and Oswestry Disability Index at 3 days, 3 months, and 6 months postoperatively were not significant (*P* > .05). Although duration of surgery in the navigational robot group was significantly longer than in the fluoroscopy group (*P* > .05), the intraoperative blood loss and the intraoperative fluoroscopy times were significantly lower than in the fluoroscopy group (*P* < .05). The difference in the PHS between the 2 groups was not significant (*P* > .05). The APS in the navigation robot group was significantly higher than in the fluoroscopy group, and the rate of FJV was significantly lower than in the fluoroscopy group (*P* < .05). Compared with the traditional fluoroscopic technique, navigation robot-assisted lumbar interbody fusion with internal fixation provides less postoperative low back pain in the short term, with less trauma, less bleeding, and lower radiation exposure, as well as better APS and lower FJV, resulting in better clinical efficacy and safety.

## 1. Introduction

Lumber degenerative diseases (LDD) are one of the most common conditions in spinal surgery, and the chronic low back pain (LBP) they cause significantly reduces patients’ quality of life.^[1]^ Lumbar interbody fusion has become a classic procedure for the treatment of LDD, as it can completely decompress the spinal cord or nerve roots, correct the deformity, and utilize pedicle screw fixation to maintain good spinal stability.^[2]^ Oblique lumbar interbody fusion (OLIF) was first proposed in 2012 and has gradually been widely used in clinical practice. It enters the intervertebral space through the natural cavity between the psoas major muscle and the large abdominal blood vessels, and not only directly decompresses the vertebral space, but also implants a larger interbody fusion cage, which can help to better correct the spinal imbalance, and reduce the injury of paravertebral muscles, decrease the amount of blood loss, and promote the patient’s rapid recovery.^[3,4]^ Good pedicle screw internal fixation is a key factor in maintaining stability after decompression of OLIF and correction of spinal imbalance. Percutaneous pedicle screws (PPS) internal fixation is widely used in the clinic because of its small incision, less injury, and less bleeding, and how to ensure accurate and safe placement of PPS is a key concern in the clinic.^[5]^ As a result, robotics and navigation technologies have emerged, and the successful clinical use of robotics in particular has significantly improved the accuracy and safety of PPS placement.^[6]^ With the continuous development and improvement of robotics, the new generation of navigation robots combines three-dimensional (3D) optical navigation, which is expected to better improve performance and accuracy, but there are fewer studies on navigation robot-guided OLIF combined with PPS for the treatment of LDD, it is still debatable whether robot-assisted screw placement achieves a higher level of efficacy and safety than traditional technology.^[7,8]^ Therefore, this study intends to retrospectively analyze the clinical efficacy and safety of navigation robot-assisted OLIF combined with PPS and fluoroscopic freehand screw placement and to provide theoretical support for the clinical application of the robot.

## 2. Materials and methods

### 2.1. Inclusion and exclusion criteria

Inclusion criteria: patients diagnosed with LDD that had been ineffective with conservative treatment for more than 6 months and underwent OLIF combined with PPS placement; patients who chose navigational robot-assisted or fluoroscopic freehand operation for PPS placement; and patients who underwent surgical treatment for single-segment fusions.

Exclusion criteria: history of lumbar spine surgery in the operated segment; patients with multisegment LDD who underwent fusion; patients with severe osteoporosis who had cemented screw placement; patients with severe deformity of the pedicles who were unable to complete the screw placement; and patients who were unable to undergo surgical treatment due to other diseases.

### 2.2. Baseline information

According to the inclusion and exclusion criteria, 38 patients from March 2022 to May 2023 were included, all patients signed a written informed consent and received ethical approval before the start of the study (2022XE-QZRYS0061). The decompression and fusion cage implantation were accomplished using the OLIF procedure in both groups, navigation robot-assisted PPS placement was used in 16 cases, and fluoroscopic freehand PPS was used in 22 cases. Both groups of patients had poor preoperative outcomes after 6 months or more of conservative treatment and were determined to have severe LDD by imaging. The differences in gender, age, body mass index, lesion segment, and disease category between the 2 groups were collected and analyzed.

### 2.3. Surgical method

#### 2.3.1. Navigational robotic-assisted OLIF combined with PPS

The third-generation Mazor X navigation robot was used to complete the OLIF combined with PPS. Patients completed lumbar computed tomography (CT) scanning 1 day before surgery and were imported into the Mazor X navigation robot system, and the preoperative planning of the needle entry point, angle, and screw specification and direction was performed in the software to calculate the optimal trajectory for screw placement. In surgery, after the anesthesia took effect, the patient was placed in the right lateral position so that his dorsal side was about 10 cm from the edge of the surgical bed. The implantation of the lumbar interbody fusion device by OLIF was completed first, and the surgical vertebral body was identified under fluoroscopy after routine disinfection and laying of sheets, and a surgical incision of about 5 cm in length was made centered on the fusion segment. The skin and subcutaneous fascia were incised layer by layer, the external abdominal oblique, internal abdominal oblique, and transversus abdominis muscles were bluntly separated, the peritoneum was separated forwardly and the psoas major muscle was accessible, the fingers bluntly separated the anterolateral lumbar psoas interspace, the surgical vertebral body was exposed, and the vertebral space was propped up with an automatic propping device. Fluoroscopy was performed again to determine the surgical space, the sharp knife cross-cut the fibrous annulus, the rongeurs were used to remove the nucleus pulposus and fibrous annulus, and different sizes of reamers were used to deal with the intervertebral space in turn. After thorough removal of the intervertebral space to the cartilage endplates bilaterally, the appropriate size trial mold was selected, the intervertebral fusion device was implanted after the intervertebral space was cleaned with saline, the fusion device was confirmed to be well-positioned under fluoroscopy, the incision was covered with a sterile dressing after a large amount of saline rinsing. Subsequently, the robotic arm was mounted on the outside of the operating bed, and the navigation system was placed in the designated position and connected to a tracer fixed to the posterior superior iliac spine. After wrapping the robotic arm with a sterile sleeve to complete tool validation and table setting, image stitching and 3D modeling techniques were used to define the safe distance between the robot and the patient during the operation, as well as to establish a no-fly zone for the robotic arm. Correlate the navigation reference system to the robotic arm reference system via Snapshot snapshot to enable navigational function. Images from intraoperative orthoptic and oblique fluoroscopy were again cross-modally aligned with the preoperative data, using the anchor channel of the Mazor X robot to complete the visualized screw placement under navigation.

**Figure 1.**
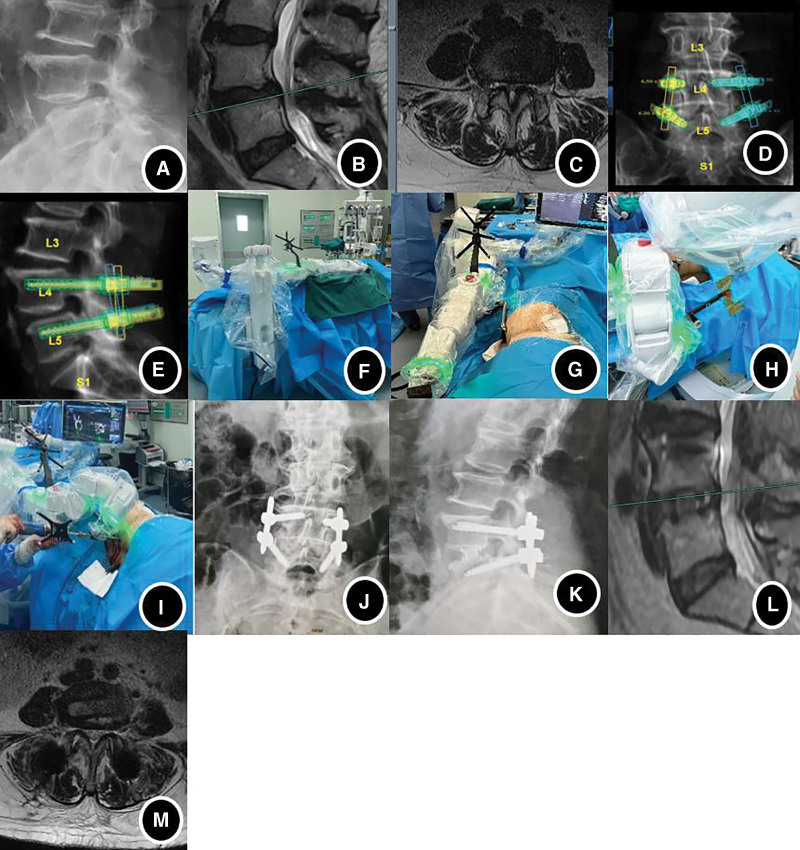
Navigational robot-assisted single-segment OLIF combined with PPS. (A) Preoperative lumbar X-ray suggesting L4 vertebrae spondylolisthesis. (B, C) Preoperative MRI suggesting L4/5 segmental spinal stenosis. (D, E) Preoperative CT imported into the Mazor X system and preoperative screw placement planning and simulation. (F) Establishment of the Mazor X navigational robotic system and positional placement. (G) After completion of the OLIF, the navigational reference system was correlated with the robotic arm’s reference system to enable the navigational function. (H) Intraoperative fluoroscopy and cross-modal alignment of preoperative CT data. (I) Full visualization of screw placement under 3D optical navigation guidance. (J, K) Postoperative lumbar X-rays suggesting the spondylolisthesis was effectively reduced and fixed. (L, M) Postoperative lumbar MRI suggesting a significant increase in the diameter of spinal canal. 3D = three-dimensional, CT = computed tomography, MRI = magnetic resonance imaging, OLIF = oblique lumbar interbody fusion.

After screw placement, 4 pedicle screws were connected with 2 titanium rods and were locked with bilateral pressure. The pedicle screws were well-positioned under fluoroscopy, rinsed with large amounts of saline, and bandaged with sterile dressings after suturing layer by layer (Fig. 1).

#### 2.3.2. Traditional fluoroscopic OLIF combined with PPS

After the anesthesia took effect, the intervertebral fusion device was first implanted under the right lateral position, and the position of the intervertebral fusion device was ideally under fluoroscopy, washed with saline, and then sutured layer by layer and aseptically bandaged. Subsequently, routinely sterilized again, percutaneous bilateral pedicle screws were placed under fluoroscopy, and 4 posterior pedicle screws were connected with 2 spinal titanium rods and were locked with pressure bilaterally. The position of pedicle screws was good under fluoroscopy, then closed layer by layer and wrapped up with aseptic dressings after being washed out with saline.

### 2.4. Perioperative management

Cefazolin sodium 2.0 g was injected intravenously 30 minutes before and 24 hours after the operation to prevent infection. On the 2nd postoperative day, the patients were asked to wear waist cuffs to go down to the ground for moderate activities, and thereafter gradually increase the activities, and the time of wearing waist cuffs was not more than 3 months, and avoiding bending over and bearing weight too much for 3 months after the operation. X-ray and CT of the lumbar spine were performed 1 week after surgery to observe the position of the fusion device and screw placement.

### 2.5. Outcome indicator

#### 2.5.1. Clinical and perioperative indicators

We followed up and compared the visual analog scale (VAS) of the lower back and legs in the 2 groups to assess the degree of pain improvement before the operation, 3 days, 3 months, and 6 months after the operation, and the Oswestry Disability Index was used to assess the degree of functional improvement in the 2 groups before surgery, 3 days, 3 months, and 6 months after surgery. Differences in perioperative indicators such as intraoperative blood loss (IBL), duration of surgery (DS), intraoperative fluoroscopy times (IFT), and postoperative hospital stay were recorded and compared between the 2 groups.

#### 2.5.2. Imaging indicators

Lumbar spine CT was performed preoperatively and 1 week postoperatively. The accuracy of pedicle screws (APS) was evaluated on lumbar CT according to the Gertzbein and Robbins^[9]^ grading: grade A is no cortical penetration, grade B is cortical penetration distance ≤2 mm, grade C is 2 mm ≤cortical penetration distance <4 mm, grade D is 4 mm ≤cortical penetration distance <6 mm and grade E is cortical penetration distance ≥6 mm, grade A was considered as excellent position, grade B indicated acceptable screw position, and grades C–E were considered as unacceptable screw position. The rate of facet joint violation (FJV) was assessed according to the Babu et al^[10]^ grading: grade 0 is screw not in the facet joint, grade 1 is screw lateral but not in the facet joint, grade 2 is facet joint penetration <1 mm, and grade 3 is facet joint penetration >1 mm or movement within the facet joint. If a deviation is detected during nail placement and corrected promptly, the result is not a nail placement deviation.

### 2.6. Statistical analysis

SPSS26.0 was used for statistical analysis, and the measurement information that conformed to normal distribution was expressed as $\bar{x}\pm s$, and the comparison between 2 groups was made by 2 independent samples *t* test. Count data were expressed as frequency or rate, and comparisons between the 2 groups were made using the $x^{2}$ test. The Mann–Whitney *U* nonparametric test was used to compare the APS and the rate of FJV between the 2 groups. *P* < .05 was used to indicate that the difference was significant.

## 3. Results

### 3.1. Baseline materials

Patients in both groups completed the surgery and were followed up for 6 months. The differences between the 2 groups in gender, age, body mass index, fusion segment, and disease category were not significant (*P* > .05), and the baseline data of the 2 groups are shown in Table 1.

**Table 1:** Comparison of baseline data between the 2 groups of patients.

	Navigational robot group (n = 16)	Fluoroscopy group (n = 22)	*t*/$x^{2}$	*P*
Gender	16	22	0.874	.350
Male	9	9		
Female	7	13		
Age (yr)	65.13 ± 8.37	62.55 ± 7.76	0.979	.334
BMI (kg/m^2^)	24.49 ± 3.99	25.79 ± 3.46	−1.076	.289
Fusion segment			0.148	.986
L2/3	1	1		
L3/4	2	3		
L4/5	10	13		
L5/S1	3	5		
Disease category			1.114	.573
Lumbar disc herniation	3	4		
Lumbar spondylolisthesis	7	13		
Lumbar spinal stenosis	6	5		

BMI = body mass index.

### 3.2. Clinical efficacy and perioperative indicators

The VAS-back in the navigational robot group was significantly lower than in the fluoroscopy group at 3 days postoperatively (*P* < .05). However, the difference between 3 and 6 months postoperatively was not significant (*P* > .05). The differences between the VAS-leg and Oswestry Disability Index were not significant between the 2 groups at 3 days, 3 months, and 6 months postoperatively (*P* > .05). The DS in the navigational robot group was significantly longer than the fluoroscopy group (*P* < .05), but the IBL and IFT were significantly lower than the fluoroscopy group (*P* < .05). The difference between the postoperative hospital stay between the 2 groups was not significant (*P* > .05). One case of postoperative lower limb pain with numbness occurred in each of the 2 groups, which was relieved after 2 weeks of oral nutritive nerve and nonsteroidal anti-inflammatory drugs, and no other complications such as incision infection, cerebrospinal fluid leakage, and dural tear occurred in both groups. The clinical efficacy and perioperative indexes of the 2 groups are shown in Table 2.

**Table 2:** Comparison of clinical efficacy and perioperative indicators between the 2 groups.

	Navigational robot group (n = 16)	Fluoroscopy group (n = 22)	*t*/$x^{2}$	*P*
VAS-back				
Preoperative	5.75 ± 1.24	6.27 ± 1.58	−1.10	.279
Postoperative 3st day	2.19 ± 0.66	3.18 ± 1.14	−3.127	.003
Postoperative 3 mo	1.81 ± 0.66	2.27 ± 0.98	−1.623	.113
Postoperative 6 mo	1.63 ± 0.72	1.45 ± 0.91	0.620	.539
VAS-leg				
Preoperative	6.75 ± 1.53	6.41 ± 1.37	0.722	.475
Postoperative 3st day	2.38 ± 0.81	2.14 ± 1.08	0.744	.462
Postoperative 3 mo	1.44 ± 0.73	1.59 ± 0.85	−0.581	.565
Postoperative 6 mo	1.25 ± 0.68	1.18 ± 0.85	0.264	.793
ODI (%)				
Preoperative	65.06 ± 9.45	61.95 ± 11.54	0.883	.383
Postoperative 3st day	26.31 ± 3.24	28.05 ± 8.87	−0.744	.462
Postoperative 3 mo	19.56 ± 2.68	18.14 ± 3.33	1.411	.167
Postoperative 6 mo	14.44 ± 3.76	13.41 ± 2.68	0.985	.331
DS (min)	158.75 ± 23.59	129.86 ± 18.56	4.226	<.001
IBL (mL)	89.81 ± 24.99	117.27 ± 20.87	−3.685	.001
IFT	13.31 ± 3.66	48.45 ± 8.19	−15.99	<.001
PHS (d)	7.13 ± 3.12	7.55 ± 3.50	−0.382	.704

DS = duration of surgery, IBL = intraoperative blood loss, IFT = intraoperative fluoroscopy times, ODI = Oswestry Disability Index, PHS = postoperative hospital stay, VAS = visual analog scale.

### 3.3. APS and FJV

A total of 64 screws were placed in the navigational robot group, of which 60 were A-grade screws, 3 were B-grade screws, 1 was C-grade screw, and there were no D-grade or E-grade screws, with an acceptable rate of screw placement of 98.4%. In the fluoroscopy group, a total of 88 screws were placed, of which 70 were grade A screws, 14 were grade B screws, 3 were grade C screws, 1 was grade D screw, and there was no grade E screw, with an acceptable rate of screw placement of 95.5%. The APS in the navigational robot group was significantly higher than the fluoroscopic group (*P* < .05). In terms of FJV, the navigational robot group had 61 grade 0 screws, 2 grade 1 screws, 1 grade 2 screw, and no grade 3 screws, for FJV rate of 4.7%. The fluoroscopy group had 71 grade 0 screws, 11 grade 1 screws, 5 grade 2 screws, 1 grade 3 screw, and a 19.3% rate of FJV. The rate of FJV in the navigational robot group was significantly lower than in the fluoroscopy group (*P* < .05). The difference between the APS and the rate of FJV in the 2 groups is shown in Table 3.

**Table 3:** Comparison of APS and FJV rates between the 2 groups.

	Navigational robot group (n = 16)	Fluoroscopy group (n = 22)	*Z*	*P*
APS			−2.441	.015
A	60 (93.8%)	70 (79.6%)		
B	3 (4.6%)	14 (15.9%)		
A + B	63 (98.4%)	84 (95.5%)		
C	1 (1.6%)	3 (3.4%)		
D	0 (0%)	1 (1.1%)		
E	0 (0%)	0 (0%)		
FJV			−2.626	.009
Grade 0	61 (95.3%)	71 (80.7%)		
Grade 1	2 (3.1%)	11 (12.5%)		
Grade 2	1 (1.6%)	5 (5.7%)		
Grade 3	0 (0%)	1 (1.1%)		

APS = accuracy of pedicle screws, FJV = facet joint violation.

## 4. Discussion

Internal fixation with pedicle screws is one of the landmark inventions in spinal surgery, which is a key factor in the success of lumbar fusion with internal fixation.^[11]^ As spinal surgery enters the era of minimally invasive surgery, the placement of pedicle screws has evolved from open screw placement to PPS, which significantly increases minimally invasiveness, however, it also increases the risk and difficulty of screw placement.^[12]^ Due to the proximity of the pedicle to the spinal canal, nerve roots, and blood vessels, incorrect screw placement may lead to a series of complications such as nerve and blood vessel injuries, cerebrospinal fluid leakage, dural tear, and failure of internal fixation, which may significantly reduce the efficacy and safety of the surgery.^[13]^ The accuracy and safety of the PPS under fluoroscopy are mainly dependent on the experience of the operator and repeated fluoroscopy, but the rate of strict misalignment is still as high as 4.1%, which significantly increases the rate of complications and radiation exposure of the doctor and patient.^[14]^ With orthopedic robots are gradually applied to spinal surgery and are considered to have good efficacy and safety, which is an important adjunctive technology for PPS.^[15]^ The introduction of 3D optical navigation, which integrates navigation technology and robotics on the same platform, provides real-time image guidance for intraoperative screw placement, further increasing accuracy and safety.^[7]^

In this study, we found that the VAS-back in the navigational robot group was significantly lower than in the fluoroscopy group at 3 days postoperatively, which may be because the optimal point of entry needs to be determined artificially when placing the screws under fluoroscopy, and thus it is difficult to avoid repeatedly adjusting the entry position. Repeated puncture of local tissues and anchoring of the needle entry point, may significantly increase local muscle detachment and injury, leading to more extensive local vascular and nerve damage.^[16]^ Because of preoperative planning and intraoperative alignment, navigation robot-assisted screw placement eliminates the need for artificial selection of the needlepoint, and can complete 1-time precise screw placement without repeated adjustments, resulting in less damage to the local soft tissues and lower invasiveness.^[17]^ Therefore, fluoroscopic screw placement was more traumatic than the navigational robot, and thus the LBP was more pronounced in the early postoperative period, whereas over time, the local tissue damage was well repaired under both groups, and the LBP was significantly relieved, and thus the difference between the VAS-back at 3 and 6 months postoperatively was not significant. Although our study had a shorter follow-up period, Cui et al^[16]^ came to a similar conclusion with a 2-year follow-up, in which they found that robot-assisted screw placement resulted in significantly lower VAS scores than freehand screw placement at 3 days postoperatively and that this difference disappeared at the 6-month to 2-year postoperative follow-up. Similarly, Wang et al^[18]^ found that the long-term efficacy of robot-assisted screw placement was comparable to that of freehand screw placement under fluoroscopy by 2-year follow-up, both of which resulted in effective pain relief and functional recovery. Therefore, robot-assisted screw placement provides more effective early pain relief than conventional screw placement, but both have similar long-term outcomes.

Repeated fluoroscopy during screw placement is an important safeguard for freehand PPS so that the needlepoint and direction can be adjusted in time to maximize the safety of screw placement. However, it will inevitably increase the radiation exposure of the patient and operator. In addition, repeated fluoroscopy and screw adjustment will also lead to varying degrees of fatigue, which may affect the accuracy of screw placement.^[19]^ Robot-assisted techniques can effectively overcome this disadvantage of freehand screw placement, especially with the advent of navigational robots, which assist in screw placement through 3D images created by optical navigation, eliminating the need for repetitive fluoroscopy and adjustment of the screws and effectively reducing radiation exposure and operator fatigue.^[20]^ At the same time, the navigation robot has less IBL and shorter screw placement time due to a more accurate entry point that does not require repeated adjustments and significantly decreases damage to the local soft tissue and bone surface at the entry point.^[21]^ In our study, IBL and IFT in the navigational robot group were significantly lower than in the fluoroscopy group, confirming that navigational robotic-assisted screw placement is less damaging, and significantly reduces radiation exposure, resulting in a higher level of safety, which is similar to the results of several recent studies.^[16,22]^

In contrast to the findings of Asada et al,^[22]^ we found that the DS in the navigated robot group was significantly longer than in the fluoroscopy group, which may be significantly related to the operator learning curve and the robot’s intraoperative preparation. Since Huntsman et al^[23]^ reported robot-assisted single-position OLIF combined with PPS for lumbar interbody fusion in 2020, several recent studies have concluded that it results in good efficacy and significantly reduces operative time and improves safety.^[24,25]^ In this study, we also used a single position for robot-assisted screw placement, which avoids position switching and secondary disinfection of the operative area, thus being able to reduce the risks associated with anesthesia and DS prolongation. Fluoroscopic freehand screw placement, on the other hand, requires a position change from lateral to prone after implantation of the fusion device, which not only prolongs the DS but also increases the risk of anesthesia. In addition, unlike conventional procedures, the robot is equipped with an intraoperative navigation and fluoroscopy system, which significantly shortens the surgical process and DS by eliminating the need to change positions due to fluoroscopy, even when screws are placed in a single position. Although robotic-assisted techniques simplify the screw placement process, additional intraoperative planning and surgical preparation will lead to a prolongation of the overall DS, while with the increasing experience of the operator, the DS consumed by the robotic-assisted PPS will be gradually shortened and reach a plateau, obtaining a DS similar to or even shorter than the freehand screw placement.^[26]^

The APS and FJV of PPS are important metrics for assessing the safety of pedicle screws. Most studies concluded that robot-assisted screw placement was significantly more accurate than freehand screw placement under fluoroscopy, but a few studies still came to the opposite conclusion, suggesting that robot-assisted screw placement has a higher rate of misalignment.^[27]^ Real-time navigation techniques are thought to improve APS and reduce the incidence of FJV in PPS, significantly outperforming freehand screw placement under fluoroscopy.^[28]^ Hiyama et al^[29]^ implemented a PPS internal fixation technique in the lateral position using CT navigation and found that the deviation rate of lumbar screw placement was only 1.9% and concluded that patient prognosis could be improved by improving APS. This shows that navigation-assisted screw placement is superior to fluoroscopic freehand screw placement and can be performed in the lateral recumbent position, and when it is combined with robotics it is possible to accomplish the OLIF combined with PPS technique in a single position and further improve APS and reduce FJV. In our study, we found that the grade A screws with robotic assistance was 93.8%, which was similar to the accuracy reported with the Mazor X Stealth technology (94.1%), and was significantly higher than the accuracy of freehand screw placement under fluoroscopy (79.6%).^[7]^ Similarly, Khan et al^[30]^ reported an APS of 98.7% for the Mazor X system, which shows that the use of navigational robotics significantly improves the APS of the PPS. FJV is one of the most common complications of lumbar fusion and internal fixation, and its hyperplastic degeneration not only leads to the occurrence of degeneration in neighboring segments, but also has the potential to affect the fusion rate, which can lead to the development or exacerbation of LBP, and it is one of the main reasons for reoperation.^[31]^ Studies have shown that there is no significant difference between freehand screw placement under fluoroscopy in the lateral and prone positions, but there is a higher incidence of grade 2 screws for screw placement in the lateral position.^[32]^ The incidence of FJV was significantly reduced when navigation or robot-assisted techniques were used in PPS internal fixation.^[33,34]^ The FJV of PPS under navigational robot assistance in our study was only 4.7%, which was significantly lower than the 19.3% of PPS under fluoroscopy, suggesting that PPS under navigational robot assistance is safer and further reduces the risk of long-term complications, such as neighboring segment degeneration and low fusion rate, in accordance with the results of Meta-analysis by Li et al.^[35]^

Cost-effectiveness has been one of the hot topics of interest in robot-assisted technology, and it is generally recognized that robots have a high initial cost and a range of other costs such as service, maintenance, and training fees.^[36]^ Passias et al^[37]^ concluded that robotic-assisted techniques have higher complications and costs compared to conventional procedures. However, Menger et al^[38]^ concluded that robotic-assisted techniques were able to significantly reduce postoperative revision rates, infection rates, and shorten postoperative hospitalization and DS compared to conventional fluoroscopic techniques, thus providing better cost-effectiveness. Garcia et al^[39]^ also found through a cost-effectiveness analysis that the robot-assisted technology was estimated to cost $21,546.8 while the non-robot-assisted technology was estimated to cost $22,398.8, indicating that the robot-assisted technology had higher benefits and lower costs than the non-robot-assisted technology. Therefore, there is still much controversy about the cost-effectiveness of robot-assisted technologies, and more thorough research is still needed to further assess their cost-effectiveness. With the development of the times and the progress of science and technology, we believe that robot-assisted technology will have lower costs and higher effects with higher safety and accuracy in the future.

## 5. Limitations

Our study still has some limitations. First, this is a retrospective study, which may lead to the occurrence of selection bias, thus reducing the reliability of the study data; second, the small sample size included is also one of the shortcomings of our study, which may lead to the insufficient credibility of the results; third, the follow-up period of this study is relatively short, which is only 6 months, resulting in the lack of data on the long-term efficacy and complications of the follow-up. A large-sample and multi-center clinical studies are still needed to confirm the advantages of the navigational robots’ application.

## 6. Conclusion

Compared with freehand screw placement under fluoroscopy, navigation robot-assisted screw placement not only significantly reduces the short-term postoperative LBP, but also has the advantages of less trauma, less bleeding, fewer fluoroscopies, higher accuracy, and lower rate of FJV, which provides better clinical efficacy and safety and significantly reduces the risk of postoperative long-term complications such as degeneration of the neighboring segments and nonunion of the bone grafts.

## Author contributions

**Conceptualization:** Min Tong, Siping Zhang, Wenhao Zhang.

**Funding acquisition:** Min Tong.

**Writing—original draft:** Min Tong, Shida Li.

**Writing—review & editing:** Min Tong, Yifei Huang.

**Formal analysis:** Siping Zhang.

**Data curation:** Limin Mou.

**Methodology:** Zhenyu Dong.

**Supervision:** Rong Wang.

## References

[1] Kim HS Wu PH Jang IT. Lumbar degenerative disease part 1: anatomy and pathophysiology of intervertebral discogenic pain and radiofrequency ablation of basivertebral and sinuvertebral nerve treatment for chronic discogenic back pain: a prospective case series and review of literature. Int J Mol Sci. 2020;21:1483. 32098249 10.3390/ijms21041483 PMC7073116

[2] Zhang HQ Wang CC Zhang RJ. Predictors of accurate intrapedicular screw placement in single-level lumbar (L4-5) fusion: robot-assisted pedicle screw, traditional pedicle screw, and cortical bone trajectory screw insertion. BMC Surg. 2022;22:284. 35871659 10.1186/s12893-022-01733-6 PMC9310465

[3] Li R Li X Zhou H Jiang W. Development and application of oblique lumbar interbody fusion. Orthop Surg. 2020;12:355–65. 32174024 10.1111/os.12625 PMC7967883

[4] Park SJ Hwang JM Cho DC. Indirect decompression using oblique lumbar interbody fusion revision surgery following previous posterior decompression: comparison of clinical and radiologic outcomes between direct and indirect decompression revision surgery. Neurospine. 2022;19:544–54. 36203280 10.14245/ns.2244242.121 PMC9537844

[5] Panero I Lagares A Alén JA. Efficacy of percutaneous pedicle screws for thoracic and lumbar spine fractures compared with open technique. J Neurosurg Sci. 2023;67:462–70. 34114432 10.23736/S0390-5616.21.05332-7

[6] Shafi KA Pompeu YA Vaishnav AS. Does robot-assisted navigation influence pedicle screw selection and accuracy in minimally invasive spine surgery. Neurosurg Focus. 2022;52:E4. 10.3171/2021.10.FOCUS21526 34973674

[7] Abel F Avrumova F Goldman SN Abjornson C Lebl DR. Robotic-navigated assistance in spine surgery. Bone Joint J. 2023;105-B:543–50. 37121590 10.1302/0301-620X.105B5.BJJ-2022-0810.R3

[8] Wang C Zhang H Zhang L. Accuracy and deviation analysis of robot-assisted spinal implants: a retrospective overview of 105 cases and preliminary comparison to open freehand surgery in lumbar spondylolisthesis. Int J Med Robot. 2021;17:e2273. 33949099 10.1002/rcs.2273

[9] Gertzbein SD Robbins SE. Accuracy of pedicular screw placement in vivo. Spine (Phila Pa 1976). 1990;15:11–4. 2326693 10.1097/00007632-199001000-00004

[10] Babu R Park JG Mehta AI. Comparison of superior-level facet joint violations during open and percutaneous pedicle screw placement. Neurosurgery. 2012;71:962–70. 22843132 10.1227/NEU.0b013e31826a88c8 PMC3477296

[11] Zhang JN Fan Y He X Liu T-J Hao D-J. Comparison of robot-assisted and freehand pedicle screw placement for lumbar revision surgery. Int Orthop. 2021;45:1531–8. 32989559 10.1007/s00264-020-04825-1

[12] Fayed I Tai A Triano MJ. Lateral versus prone robot-assisted percutaneous pedicle screw placement: a CT-based comparative assessment of accuracy. J Neurosurg Spine. 2022;37:112–20. 35120316 10.3171/2021.12.SPINE211176

[13] Zhang Y Liu W Zhao J. Improving pedicle screw path planning by vertebral posture estimation. Phys Med Biol. 2023;68. 10.1088/1361-6560/ace753 37442124

[14] Han XG Tang GQ Han X. Comparison of outcomes between robot-assisted minimally invasive transforaminal lumbar interbody fusion and oblique lumbar interbody fusion in single-level lumbar spondylolisthesis. Orthop Surg. 2021;13:2093–101. 34596342 10.1111/os.13151 PMC8528977

[15] Li Y Chen L Liu Y. Accuracy and safety of robot-assisted cortical bone trajectory screw placement: a comparison of robot-assisted technique with fluoroscopy-assisted approach. BMC Musculoskelet Disord. 2022;23:328. 35387621 10.1186/s12891-022-05206-y PMC8988323

[16] Cui GY Han XG Wei Y. Robot-assisted minimally invasive transforaminal lumbar interbody fusion in the treatment of lumbar spondylolisthesis. Orthop Surg. 2021;13:1960–8. 34516712 10.1111/os.13044 PMC8528995

[17] Sun WX Huang WQ Li HY. Clinical efficacy of robotic spine surgery: an updated systematic review of 20 randomized controlled trials. EFORT Open Rev. 2023;8:841–53. 37909700 10.1530/EOR-23-0125 PMC10646522

[18] Wang L Li C Wang Z. Comparison of robot-assisted versus fluoroscopy-assisted minimally invasive transforaminal lumbar interbody fusion for degenerative lumbar spinal diseases: 2-year follow-up. J Robot Surg. 2023;17:473–85. 35788970 10.1007/s11701-022-01442-5

[19] Overley SC Cho SK Mehta AI Arnold PM. Navigation and robotics in spinal surgery: where are we now? Neurosurgery. 2017;80(3S):S86–99. 28350944 10.1093/neuros/nyw077

[20] Fu W Tong J Liu G. Robot-assisted technique vs conventional freehand technique in spine surgery: a meta-analysis. Int J Clin Pract. 2021;75:e13964. 33370470 10.1111/ijcp.13964

[21] Chen X Feng F Yu X. Robot-assisted orthopedic surgery in the treatment of adult degenerative scoliosis: a preliminary clinical report. J Orthop Surg Res. 2020;15:282. 32711566 10.1186/s13018-020-01796-2 PMC7382042

[22] Asada T Simon CZ Lu AZ. Robot-navigated pedicle screw insertion can reduce intraoperative blood loss and length of hospital stay: analysis of 1,633 patients utilizing propensity score matching. Spine J. 2024;24:118–24. 37704046 10.1016/j.spinee.2023.09.004

[23] Huntsman KT Riggleman JR Ahrendtsen LA Ledonio CG. Navigated robot-guided pedicle screws placed successfully in single-position lateral lumbar interbody fusion. J Robot Surg. 2020;14:643–7. 31625074 10.1007/s11701-019-01034-w PMC7347701

[24] Pham MH Diaz-Aguilar LD Shah V Brandel M Loya J Lehman RA. Simultaneous robotic single position oblique lumbar interbody fusion with bilateral sacropelvic fixation in lateral decubitus. Neurospine. 2021;18:406–12. 34218623 10.14245/ns.2040774.387 PMC8255773

[25] Diaz-Aguilar LD Shah V Himstead A Brown NJ Abraham ME Pham MH. Simultaneous robotic single-position surgery (SR-SPS) with oblique lumbar interbody fusion: a case series. World Neurosurg. 2021;151:e1036–43. 34033960 10.1016/j.wneu.2021.05.043

[26] Chen X Song Q Wang K. Robot-assisted minimally invasive transforaminal lumbar interbody fusion versus open transforaminal lumbar interbody fusion: a retrospective matched-control analysis for clinical and quality-of-life outcomes. J Comp Eff Res. 2021;10:845–56. 33906371 10.2217/cer-2021-0078

[27] Ringel F Stüer C Reinke A. Accuracy of robot-assisted placement of lumbar and sacral pedicle screws: a prospective randomized comparison to conventional freehand screw implantation. Spine (Phila Pa 1976). 2012;37:E496–501. 22310097 10.1097/BRS.0b013e31824b7767

[28] Hiyama A Katoh H Nomura S Sakai D Watanabe M. Intraoperative computed tomography-guided navigation versus fluoroscopy for single-position surgery after lateral lumbar interbody fusion. J Clin Neurosci. 2021;93:75–81. 34656265 10.1016/j.jocn.2021.08.023

[29] Hiyama A Sakai D Katoh H Nomura S Watanabe M. Assessing procedural accuracy in lateral spine surgery: a retrospective analysis of percutaneous pedicle screw placement with intraoperative CT navigation. J Clin Med. 2023;12:6914. 37959378 10.3390/jcm12216914 PMC10647313

[30] Khan A Meyers JE Siasios I Pollina J. Next-generation robotic spine surgery: first report on feasibility, safety, and learning curve. Oper Neurosurg (Hagerstown). 2019;17:61–9. 30247684 10.1093/ons/opy280

[31] Le X Tian W Shi Z. Robot-assisted versus fluoroscopy-assisted cortical bone trajectory screw instrumentation in lumbar spinal surgery: a matched-cohort comparison. World Neurosurg. 2018;120:e745–51. 30172976 10.1016/j.wneu.2018.08.157

[32] Hiyama A Katoh H Sakai D Tanaka M Sato M Watanabe M. Facet joint violation after single-position versus dual-position lateral interbody fusion and percutaneous pedicle screw fixation: a comparison of two techniques. J Clin Neurosci. 2020;78:47–52. 32616353 10.1016/j.jocn.2020.06.016

[33] Yson SC Sembrano JN Sanders PC Santos ERG Ledonio CGT Polly DW. Comparison of cranial facet joint violation rates between open and percutaneous pedicle screw placement using intraoperative 3-D CT (O-arm) computer navigation. Spine (Phila Pa 1976). 2013;38:E251–8. 23197012 10.1097/BRS.0b013e31827ecbf1

[34] Zhang Q Xu YF Tian W. Comparison of superior-level facet joint violations between robot-assisted percutaneous pedicle screw placement and conventional open fluoroscopic-guided pedicle screw placement. Orthop Surg. 2019;11:850–6. 31663290 10.1111/os.12534 PMC6819175

[35] Li HM Zhang RJ Shen CL. Accuracy of pedicle screw placement and clinical outcomes of robot-assisted technique versus conventional freehand technique in spine surgery from nine randomized controlled trials: a meta-analysis. Spine (Phila Pa 1976). 2020;45:E111–9. 31404053 10.1097/BRS.0000000000003193

[36] Lee YS Cho DC Kim KT. Navigation-guided/robot-assisted spinal surgery: a review article. Neurospine. 2024;21:8–17. 38569627 10.14245/ns.2347184.592 PMC10992634

[37] Passias PG Brown AE Alas H. A cost benefit analysis of increasing surgical technology in lumbar spine fusion. Spine J. 2021;21:193–201. 33069859 10.1016/j.spinee.2020.10.012

[38] Menger RP Savardekar AR Farokhi F Sin A. A cost-effectiveness analysis of the integration of robotic spine technology in spine surgery. Neurospine. 2018;15:216–24. 30157583 10.14245/ns.1836082.041 PMC6226125

[39] Garcia D Akinduro OO De Biase G. Robotic-assisted vs nonrobotic-assisted minimally invasive transforaminal lumbar interbody fusion: a cost-utility analysis. Neurosurgery. 2022;90:192–8. 35023874 10.1227/NEU.0000000000001779

